# The Effect of an Evaluative Label on Consumer Perception of Cheeses in Hungary

**DOI:** 10.3390/foods9050563

**Published:** 2020-05-02

**Authors:** Zoltán Szakály, Mihály Soós, Nikolett Balsa-Budai, Sándor Kovács, Enikő Kontor

**Affiliations:** 1 Institute of Marketing and Commerce, Faculty of Economics and Business, University of Debrecen, Böszörményi Str. 138, 4032 Debrecen, Hungary; szakaly.zoltan@econ.unideb.hu (Z.S.); soos.mihaly@econ.unideb.hu (M.S.); budai.nikolett@econ.unideb.hu (N.B.-B.); 2 Institute of Sectorial Economics and Methodology, Faculty of Economics and Business, University of Debrecen, Böszörményi Str. 138, 4032 Debrecen, Hungary; kovacs.sandor@econ.unideb.hu

**Keywords:** nudge, evaluative label, health-halo effect, consumer perception, cheese

## Abstract

Traditional strategies (such as education, economic incentives or prohibitions) targeted at altering dietary habits only influence health-conscious consumers. Less health-conscious consumers are less capable of self-regulatory behavior, therefore they are more likely to be influenced through perception. The present study aimed to examine how external cues such as labeling affect the consumer’s perception of foods. The paper includes a case study based on an experiment. In the experiment the same cheese was tested with four different types of labeling (labeled “conventional”, “low salt”, “low fat” and “low salt and low fat”). It was found that the health halo effect worked in the case of cheese testing. In spite of all the samples being identical, the healthy samples were associated with considerably less sensory pleasure. The use of labels by the producers resulted in exactly the opposite effect to that intended. The experiment confirmed the efficiency of the application of this type of behavior-oriented nudge.

## 1. Introduction

The past few decades have seen a dramatic increase in non-communicable diseases worldwide and the mortality rates related to them have been the highest for a long time [1]. In its annual report, ILSI Europe (2018) published exact figures concerning this issue. In Europe alone, 11–26 million people suffer from food allergy or food intolerance; on a global scale the body mass of 1.9 billion people exceeds the normal index, 650 million of whom are obese, while 2 billion people lack vitamins and minerals in their daily diet. The situation is no better among school age children: it is estimated that 268 million children will be overweight by 2025, with 91 million of them being obese.

Research in this field soon revealed that it is primarily the changes in the way of life of humankind that are to blame for the spread of non-communicable diseases [2]. Despite the increasing personal motivation, the accelerated pace of life, massive levels of environmental pollution, stress and the sedentary lifestyle induced by market competition, people’s dietary habits of a thousand years have remained unchanged. The rapid global spread of chronic diseases is the direct result of nutrition not being adjusted to the altered way of life. All the findings shown above indicate that nutrition and diet play a significant role in preserving excellent health, although the role of other factors, such as regular physical activity, healthy ageing, the cleanliness of the environment, a balanced lifestyle, the comprehensive role of culture and tradition are also crucial [2].

It is of major importance in terms of nutritional behavior that factors driving eating decisions are not always conscious ones, and rationality is often neglected. People are influenced by several drivers unknowingly, only at a perceptual level. The majority of food consumption can be considered an unconscious activity, since it is not driven by hunger or nutritional needs, but by environmental cues such as “family and friends, packages and plates, names and numbers, labels and lights, colors and candles, shapes and smells, distractions and distances, cupboards and containers” [3] (p. 1). Unconscious eating endangers the health of consumers, since they believe that their eating behavior is controlled by hunger, by their mood or by their dietary preferences, whereas in reality they are influenced by environmental cues and do not act with appropriate self-awareness [3,4]. Therefore, it seems worthwhile to explore the causes and mechanisms that lead to mindless eating and eventually to obesity.

### 1.1. Literature Review

There is always self-regulatory behavior behind conscious food choice decisions and mindful nutrition, which also involves responses to external environmental stimuli. In order to achieve their own goals and needs, individuals continuously adapt to changes in the environment, and if necessary, they make meaningful changes in certain activities as well, and this may also involve conscious alterations in behavioral patterns in terms of nutrition [5,6].

In regards to controlling the amount of food consumed, self-regulation refers to efforts that regulate and maintain the appropriate selection and amount of food consumption in such a way that the individual resists the temptations and avoid situations, which may stimulate overconsumption [7]. After screening the literature, Poelman et al. (2014) identified 32 strategies for behavioral changes based on the mechanism of self-regulation. These strategies were proved to be effective and useful in weight management, although primarily among people with a normal body mass index [7].

Food purchase and food consumption in the adequate quantity and quality is closely linked to consumers’ involvement with foods. If involvement with food is defined as the importance, the hedonic value and the risk of foods in an individual’s life, it can be assumed that the degree of involvement with foods is likely to differ between individuals. Highly involved consumers tend to look for diversity, they consider several selection criteria, and their sophisticated taste physiology provides them with a higher sense of pleasure in the meal [8,9,10,11,12]. This would also imply that they are considerably more capable of making distinctions between foods merely on the basis of the sensory properties of the foods themselves. Naturally, there are several other factors that influence the importance of a product, such as product category, expectations and motivation or risk factors arising in connection with the food, including social, health and functional risks [8,13,14,15,16].

Relatively little is known about how external environmental stimuli (environmental cues and incentives) influence the selection and the amount of food consumed [17,18]. Food consumption is also unknowingly influenced by external environmental cues such as the number of items in an assortment, which we can choose from, or the eating behavior of our companions during the meal, which may serve as a reference point for a person to gauge how much to eat and drink. Similarly, larger packages, serving bowls and plates can all contribute to an increase in food consumption by as much as 10%–45%, according to a research study [3,19]. Every single day, factors such as the visibility, size and accessibility of food increase the ever-growing rate of obesity in developed countries [20,21].

The points described above indicate that some of the factors that drive eating decisions cannot be explained by purely reason-based models as is frequently suggested by many. The effects of the drivers may appear at a perceptual and preconscious level; therefore more attention should be paid to this area, i.e., an examination should be made of the way in which external cues, perceived unknowingly, influence eating behavior.

#### Behavior Change Strategies

There are two types of strategies available for changing eating habits [22]. One type is represented by traditional strategies for behavior change, which basically involve interventions by policies at the state, sectoral and non-governmental level. The other group comprises innovative behavior change strategies, which can rather be implemented at a corporate or individual level.

##### Traditional behavior change strategies

Traditional behavior change strategies include awareness-raising (education) [23], economic incentives and prohibitions [24]. Public health campaigns, aimed at reducing risk behavior and strengthening preventive health behavior among the population are a good example of awareness-raising (examples include social campaigns such as the Stop Salt program, Food Only Wisely! or Food Safety for All!). Economic incentives include different taxes and subsidies. Perhaps in Hungary the best known among them is the Public Health Product Tax (abbreviated as NETA), according to which the first domestic sale of products with different sugar, salt and caffeine content and also that of alcoholic beverages, is subject to tax.

Several research studies, however, have pointed out that the use of traditional behavior change strategies and tools are of low effectiveness, in that they have failed to substantially improve the health of the population and have failed to reduce the consumption of “non-useful” foods [3,19,22].

##### New behavior change strategies

Novel behavior change strategies are basically oriented towards two main target groups. One of the segments consists of the so-called health-conscious consumers, who show stability in their dietary behavior. Consumers in this segment already possess the ability to control their own actions, and preventive health behavior and conscious nutrition have become a natural part of their lives.

However, the less health-conscious, emotional or impulsive eaters are often incapable of self-regulatory behavior, therefore they tend to be influenced primarily by the organoleptic properties of food, and they can sooner be addressed through perception and cognition. Consequently, researchers have recently become increasingly interested in the so-called nudge interventions. Nudges are defined by Thaler and Sunstein (2008) as “any aspect of the choice architecture that alters people’s behavior in a predictable way without forbidding any options or significantly changing their economic consequences. Putting fruit at eye level counts as a nudge; banning junk food does not” [25] (p. 6).

According to Thaler and Sunstein (2008), the basis of the nudge approach is that while it is difficult to change consumer habits, altering certain elements in the environment in which consumers make decisions may yield satisfactory results. Nudging changes to the environment in such a way that it provides consumers with certain (healthy) choice options [25].

Healthy eating nudges encompass several varieties, and numerous examples to classify them can be found in the literature. Cadario and Chandon (2019) state that research on healthy eating nudges has so far been carried out within two frameworks, based either on preventive (intervention) instruments (such as a label, or plate size), or on the hypothesized mechanisms of action (e.g., attention vs. social norm) [26]. The authors are of the opinion that the distinctions have become finer over the years and the issue has been approached from a variety of perspectives by many researchers. Following the classical tripartite classification of mental activities, the authors themselves distinguish three types of nudges: “Cognitively Oriented Interventions (“Descriptive nutritional labeling”, “Evaluative nutritional labeling”, “Visibility enhancement”)—that seek to influence what consumers know, Affectively Oriented Interventions (“Hedonic enhancement”, “Healthy eating”)—that seek to influence how consumers feel without necessarily changing what they know, and Behaviorally Oriented Interventions (“Convenience enhancement”, “Size enhancement”)—that seek to influence what consumers do without necessarily changing what they know or how they feel” [26] (p. 2).

Ly et al. (2013) categorize nudge interventions into four dimensions: (1) Boosting self-control vs. Activating a desired behavior, (2) Externally-imposed vs. Self-imposed, (3) Mindful vs. Mindless and (4) Encourage vs. Discourage. By combining these four dimensions they identified 12 nudge types, which drive consumers to make better decisions in different ways [27]. The ethical aspects of interventions were also taken into account by Blumenthal-Barby and Burroughs (2012), based on which the application options were divided into six categories [28]. This subdivision was followed by Wilson et al. (2016) in their review, where the efficiency of each nudge technique was evaluated with regard to the shift towards healthier dietary habits. In their assessment, “priming” (visibility, accessibility and availability) and “salience” (traffic light labels, descriptive labels and taste-testing) nudges were found to show a consistent positive relationship with the choice of healthy foods [29]. Hollands et al. (2013, 2017), instead of the initial three types of nudges, distinguish 18 categories in their latest TIPPME typology (placement/properties × intervention focus) [30,31].

Several reviews have been published that focus on specific areas. Broers et al. (2017, 2019), and Nørnberg et al. (2015) examined fruit and vegetable consumption [32,33,34]. Skov et al. (2012) conducted research on self-service eating settings, Thapa and Lyford (2014) looked at school canteens, while Butcher et al. (2016) studied food position [35,36,37]. In their meta-analyses, Long et al. (2015), and Littlewood et al. (2016) examined the relationship between menu calorie labeling and calories/energy ordered or purchased, while Cecchini and Warin (2016) examined the role of food labeling [38,39,40].

The majority of systematic reviews (including [26,29,33,41]) conclude that nudge-type interventions have a positive effect on changing dietary habits; however, almost all of them point out that the results cannot be generalized, and further investigations are required to confirm the actual success of each type of nudge.

Since it aims to assess the effects of health-related claims, the present study—based on the classification of Cadario and Chandon (2019) described above—examines the effects of evaluative nutritional labeling, a cognitively oriented nudge [26].

### 1.2. Evaluative Nutritional Labelling

Cognitively oriented interventions seek to influence primarily what consumers know. Evaluative nutritional labeling typically contains information about nutrients, or helps consumers be informed by means of color coding (e.g., red, yellow or green, depending on nutritional value) or possibly by using symbols (e.g., light, health-labels and heart logos) [26]. The same approach can be found in the study written by Sinclair et al. (2014), where it was established that labels (they use the terms contextual or interpretive labels) containing additional information (e.g., recommendations concerning daily calories, or health symbols) were more effective in helping consumers understand and use the information on the label [42]. Wansink and Chandon (2006) claim that labels provide both objective and subjective consumption signals. Portion size counts as an objective signal, while endorsed nutrition claims (e.g., a heart-friendly label), or relative nutrition claims (e.g., a low-fat label) are considered subjective signals [43].

Owing to the growing interest in balanced nutrition, health claims indicated on packaging are on the rise. However, as suggested in the review by Kontor et al. (2019), consumer acceptance of products labeled with health claims is not unconditional, and one of the most important conditions is taste [44,45,46]. The primary role of taste in consumer decisions has already been highlighted in several research studies. In these studies, taste expectations and taste experience were found to be particularly critical factors in making food choices [47,48,49]. These findings were also confirmed by Szakály (2008), pointing out that only 14% of Hungarian consumers surveyed were willing to purchase a food product if its taste did not meet their expectations [50]. Furthermore, Szakály et al. (2014) concluded that 57.4% of consumers only ate food whose taste they liked, even if the food was alleged to be less healthy [51]. Good taste is a necessary, self-defining feature of any food product, and bad taste is not acceptable for consumers in the case of healthy products, either [52,53,54].

Beside direct sensory experience (e.g., tasting), expectations play a decisive role in the perception and eventually in the acceptance of the sensory properties of food products [55,56,57]. According to Cardello and Sawyer (1992), positive expectations increase the probability of liking the given product as opposed to assessments based on neutral expectations [55]. In contrast, negative expectations reduce the probability of a pleasant sense of taste as opposed to assessments based on indifferent expectations [58].

The studies discussed below confirm that health information may function as a signal for taste, thus statements about fat and salt content, or health logos are able to alter the expected acceptance of the product. Fernqvist and Ekelund (2014) enlist several examples to prove the effect of expectations generated by health-related credence [59]. In the survey by Liem et al. (2012a) for example, the expected liking of a soup decreased when there was a reference to reduced salt content on the packaging. However, after tasting, no significant differences were found either in liking or in the perception of salinity between soups that had identical salt content, but which were labeled differently (control, reduced salt, tick and reduced salt + tick logos) [60]. The results of another experiment by Liem et al. (2012b) indicated that not only did the label “Now reduced in salt, great taste” influence the sense of taste adversely, but it also had an effect on compensatory use of salt, even leading consumers to oversalt the meal [61,62]. Kähkönen et al. (1999) carried out a survey with frankfurters and chocolate, and found that the expected intensity of the sensory properties of products with a reduced-fat label was given a lower score as compared to control groups (no claims, full-fat label) [63]. Kähkönen (2000) asked participants to taste chocolate and concluded that the information about fat reduction lowered the scores given for pleasantness, fatness, taste-intensity and meltability [64]. Norton et al. (2013), examining the impact of a “reduced-fat milk chocolate” label, found that the label had a significantly negative influence on the expected liking among the participants. In contrast, labeling did not influence the actual (post-tasting) liking of the participants or the evaluation of sensory properties [65].

In the research study by Schouteten et al. (2015), where the same cheese was tested with four different labels (“control cheese”, “cheese with reduced salt”, “light cheese” and “light cheese with reduced salt”), consumer perception was influenced by the health labels. Their results suggested that it was not only the expected liking that was influenced by the label, but that the perceived intensity of particular properties of the cheese was assessed differently depending on the label [66]. In the research the negative expectations reduced the preliminary assessment not only of liking, salt-intensity and fat flavor intensity, but they did so in terms of the emotional conceptualizations, which the participants associated with the products labeled in this way [66]. Further research has also proved that the differences between the emotional profile of foods are primarily driven by sensory organs [67,68].

There are counter-examples, however. For instance, Jo and Lusk (2018), despite the general trends, found a positive relationship between perceived healthiness and taste, while Bølling Johansen et al. (2010) found no significant relationship between liking and labeling when examining yoghurts with differing fat content [59,69,70]. Similarly, Czarnacka-Szymani and Jezewska-Zychowicz (2015) came to the conclusion that information concerning salt content did not change the acceptance of different cheeses with reduced salt content [71].

Having screened the variety of research on the topic, it can be concluded that one can agree with the findings of Liem et al. (2012b), claiming that although the aim of nutrient labels and health logos is to help consumers make healthier decisions, they may not be able to attract consumers who are more interested in taste than health. Health-conscious consumers are more likely to buy health-labeled products than those who are taste-oriented. This may cause the unexpected side effect of nutrient labels and logos promoting healthy choices. A number of consumers deliberately avoid products with healthy labels, since they expect these products to be less tasty [61].

The case study below will describe the results of our experiment, in which we attempted to find answers to research questions investigating what effect health labels have on the liking of a food product (in this case the cheese), on willingness to purchase and on perceiving certain organoleptic properties of the cheese. It must be emphasized that the aim of the experiment was to examine solely the effect of health-related labels on the expected and perceived sensory evaluation of cheeses, with the exclusion of all other factors. In order to eliminate the effects of other factors, in the case of willingness to purchase for example, we measured a kind of orientation rather than specific intention to purchase. In addition to the items listed above, a further objective of our research was of a methodical nature, since it also served to develop the experimental methodology applied.

## 2. Material and Methodology

### 2.1. Participants

In our research study altogether four focus group surveys were launched in two big Hungarian cities, Pécs and Debrecen. Professional organizers were used to help us with recruiting the focus groups. In both cities we organized two separate groups—one health-conscious and one flavor-conscious group—with 8–8 participants in each group, thus the experiment was conducted with a total of 32 participants. The degree of health and taste consciousness was determined on the basis of consumers’ self-assessment in a screener questionnaire. With regard to the topic of the focus group discussion, it was essential for us to recruit consumers who like cheese, find it important to consume cheese and do so on a regular basis, thus the screener questionnaire contained questions relating to these factors. On the one hand we excluded those who consume cheese less frequently than once or twice a week, and on the other hand, by measuring the level of liking on a scale of 1–5, we recruited respondents who fell into the top two categories (i.e., who liked cheese “very much” or “more or less”). As regards the importance of cheese, only those respondents were recruited for whom cheese—also based on a scale of 1–5—was “very important” or “more or less important”. This screening was necessary, since although there has been no research conducted on Hungarian consumers’ involvement with cheese so far, based on consumption data it can be assumed that cheese culture in Hungary is still at a low level. Compared to other European nations, while for example purchasing cheese for a Greek consumer is considered to be a means of self-expression [72], Trappista cheese constitutes 43% of all the cheese purchases in Hungary. Additionally, Hungarians typically choose only from one or two types of cheese a week, and the annual consumption is 14.2 kg per capita, which can be regarded as extremely low [73]. When recruiting consumers showing higher involvement, we took into account the recommendations of Bell and Marshall (2003), who claim that in order to ensure the efficiency of experiments on foods, it is necessary to select participants who have an adequate level of involvement [8].

The sociodemographic characteristics of the health-conscious group were as follows: at least middle income, not lower than secondary education, 4 participants younger than 40, 4 participants older than 40 years of age, 3 males (single-person household or married) and 5 females (single-person household or married). The background of the flavor-conscious group differed from that of the other group solely in the number of females and males (5 males and 3 females). When assembling the socio-demographics, we relied on the findings of the international literature. A good review on this is provided by Kontor et al. (2019) and Soós (2014) in his dissertation [44,74]. All the group discussions were conducted by the same qualified moderator who had appropriate expertise in the fields of both marketing and group dynamics.

### 2.2. Procedure

The thematic questions of the focus group scenario were as follows: motivations to purchase, emotional mapping, the influence of evaluative labels on the assessment of the taste of Trappista cheese and the attributes of the products. From among the questions listed above, the results of “The influence of evaluative labels on the assessment of the taste of Trappista cheeses and the attributes of the products” are discussed.

Consumers were asked to test Trappista cheese of the same composition, but with 4 different types of informative (evaluative) labeling. The four labels included Trappista cheese (without health claims); Trappista cheese—20% lower salt content; Trappista cheese—20% lower fat content and Trappista cheese—20% lower salt and 20% lower fat content. Before the survey, respondents were read the following text: “Taking into account health trends, a domestic manufacturer, beside the traditional sliced Trappista cheese, has developed three types of cheese of different composition: reduced added salt, reduced fat, reduced fat and reduced added salt.”. However, consumers were not told that the composition of cheeses, and therefore their natural palatability and taste, were identical in all the four cases. Our aim was to find out whether the participants in the focus group would feel any difference in the liking of the product and in the defining properties (saltiness and fat intensity), and also to see what effects all these would have on the willingness to purchase.

The experiment was conducted in the following steps:

First, respondents were asked to express their first thoughts and impressions with regard to the four, differently labeled cheese types. Our aim was to get information about the issue in question first by using free associations, without the constraints of a questionnaire.

In the second part of the experiment participants were required to complete a questionnaire before tasting (pretasting expected evaluation), on each of the four different labels, where they were expected to rate, using a 7-point Likert scale, liking (1—extremely dislike; 7—extremely like); salt intensity (1—not salty at all; 7—extremely salty); fat flavor intensity (1—not fatty at all; 7—extremely fatty) and willingness to purchase (1—not willing to purchase at all; 7—extremely willing to purchase; Appendix A). These questions and scales were based upon Liem et al.’s (2012a) and Schouteten et al.’s (2015) research studies.

Finally, in the third phase, the cheese samples with the different labels were tasted one after the other and all four of them were immediately evaluated by the respondents according to the same categories as before testing: liking, salt intensity, fat flavor intensity and willingness to purchase (post-tasting perceived evaluation; Appendix B). The order of samples for each group was as follows: Trappista cheese (without health claims); Trappista cheese—20% lower salt content; Trappista cheese—20% lower fat content and Trappista cheese—20% lower salt and 20% lower fat content. Obviously, the cheese samples were identical in the case of each label (conventional, 6 cm × 8 cm, sliced cheese); however, respondents were not aware of this fact.

### 2.3. Statistical Analyses

Several statistical techniques were applied for the analysis of the data: descriptive statistics (mean, median, mode, standard deviation and relative standard deviation), the Scheirer–Ray–Hare (SRH) test, Freeman’s theta statistics and the Wilcoxon signed rank test (Z-test).

The Scheirer–Ray–Hare (SRH) test is an equivalent of the parametric two-way ANOVA test including the interaction term. It is based on ranks and suitable for any situations where ordinal data is used. The test is much more conservative and normal distribution of the data is not a requirement. The procedure of the SRH test is described by Dytham (2011) [75]. First a standard parametric ANOVA should be performed and the mean square (MS) should be calculated, which is the average of the squared differences between the observations and the grand mean. Then, the sum of the squared differences (SS) of each factor is calculated and divided by the MS value, and the SS/MS ratio for each factor can be obtained. Based on these ratios, *p*-values can be computed according to a Chi-square distribution. Pairwise comparisons between the samples were performed by Dunn’s test and the Wilcoxon signed rank test was employed for two related samples (in before and after consumption comparisons). The significance level was 5% and all the calculations have been carried out using R Studio 3.4.4 [76]. In order to calculate the Scheirer–Ray–Hare test the rcompanion package was used and for the effect size calculation Freeman’s theta [77] was calculated and reported.

Associations with regard to the four, differently labeled cheese types were analyzed and represented with a network graph by using Gephi 0.9.2.

## 3. Results

**Figure 1:**
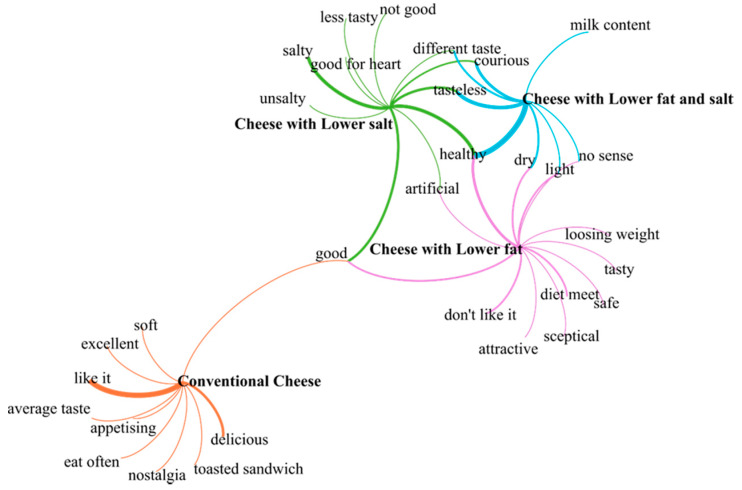
The network graph representing the associations connected to the four different labels (*N* = 32).

In the first part of the experiment we intended to reveal the respondents’ initial thoughts and associations with regard to the four, differently labeled cheese types and to represent these associations on a network graph (see Figure 1). The network graph contained four major nodes (located in the center) representing the cheese types; the other nodes were the associations to these cheese types. The associations and the cheese types were connected through edges. The thickness of an edge indicates the strength of a relationship, as associations could appear several times with respect to a given cheese type. There were common nodes for example “good” and “healthy” that could even be related to several cheese types, therefore the edges converged in these cases.

Figure 1 clearly shows that the conventional cheese was completely separate from the other three variants. In the case of the conventional Trappista cheese, consumers fundamentally had positive associations. These were partly related to the liking of cheese, including Trappista cheese (good, delicious, I like it, appetizing, I love it)—as referred to by the thicker edges in Figure 1, partly to traditions (nostalgia), to usability (toasted sandwiches) and also to frequent consumption (we often eat Trappista cheese).

In regards to the other three cheeses with the “reduced” labels, the opinions differed substantially. In the case of the cheese with a 20% lower salt content, respondents missed tastiness, and the key issue was it being unsalty. At the same time, a number of them associated the product with healthiness, which means that reduced salt content strongly divided consumers even before making a purchase. The third observation was curiosity; respondents probably had not come across such a product type in shops yet. The cheese with 20% lower fat content tended to be referred to as being light, which was linked to healthiness in a number of cases (e.g., it is safe for me to eat, the concurrence of health and diet). Considerably less negative associations were mentioned in connection with this product as compared to the cheese with a 20% lower salt content. The common ground between the two versions, however, appeared to be the perception that they were not natural, that is, they were considered artificial. It was interesting to find that despite the more negative and more skeptical associations generated by the reduced variants, the common nodes of the conventional cheese and the reduced-salt and reduced-fat variants in the association net were “good” (Figure 1), which confirms that it is not only the good taste or familiarity of a food product (in this case, the cheese), that influences the positive judgment of today’s consumers, but even it being healthy.

The cheese with 20% lower fat and 20% lower salt content created the impression of a “flavorless” product among consumers, which applied to the perception of taste, as well. Although taste aroused curiosity among consumers, they probably could not imagine the product in real life conditions, which made them uncertain. Nevertheless, the combined reduction of two components made the consumers form the association that this product is likely to be the healthiest of all the health promoting versions, which is represented by the especially thick edge in the network of associations.

As a next step, consumers were asked to what extent they liked each product type, before and also after tasting (expected and perceived liking). The results are shown in Table 1. The value of Cronbach’s alpha after tasting was above 0.6, thus the internal consistency reliability indicates that the evaluation respondents made after tasting was more reliable (Cronbach’s alpha before tasting: 0.467, after tasting: 0.673, before and after tasting together: 0.584).

The highest liking before tasting—in accordance with the first associations—was for conventional Trappista cheese. The evaluation of the product represents a high mean with low standard deviation and relative standard deviation. As was previously expected, the reduced fat product ranked second, while the product with reduced salt, together with the reduced salt, reduced fat variation, were given the lowest ratings. The results indicate that the more compounds the product becomes reduced in, the more unfavorable the expected liking will be.

**Figure 2:**
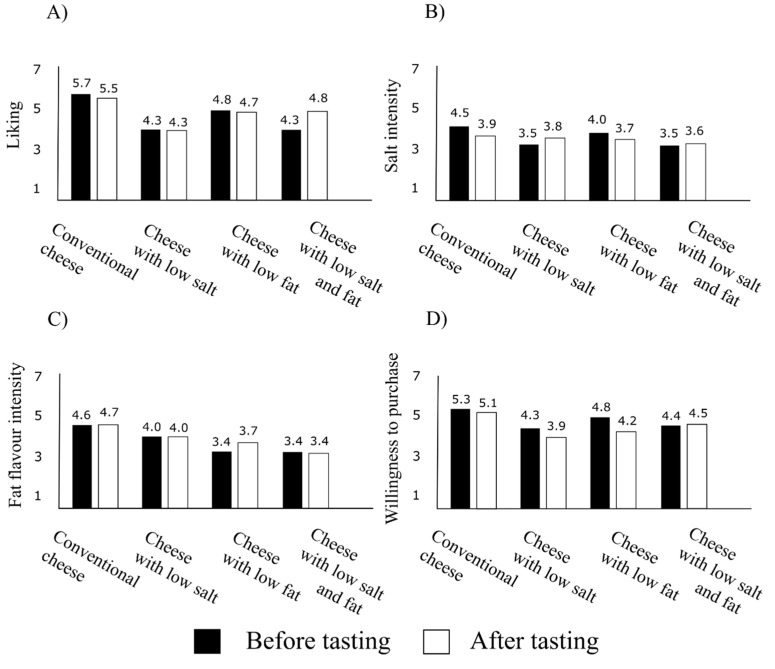
The mean of measured variables before and after tasting cheese with four different labels. (**A**) Liking; (**B**) salt intensity; (**C**) fat flavor intensity and (**D**) willingness to purchase (*N* = 32).

The question arises as to whether perceived liking after tasting would show similar tendencies as seen in expected liking. As shown in Table 1 and Figure 2a, the ranking changed significantly as compared to that experienced with expected liking. In this evaluation, it was also the conventional Trappista cheese that was given the highest liking. The mean was slightly lower than in the case of expected liking with higher standard deviation and relative standard deviation. However, the ranking was found to change completely in the case of health promoting products. A positive judgment was given to the cheese with 20% reduced fat and 20% reduced salt; it was considerably overestimated by the majority of respondents as compared to expected liking. The results suggest that consumers were surprised at the palatability of the product, since their expectations, for a lack of specific experience, had been significantly worse owing to the “flavorless” aspect. Next in the line, lagging only slightly behind, is the variant with reduced fat, while the product with 20% reduced salt ranked last in this case, as well. These findings draw attention to an important issue: in regards to product development, greater impact can be achieved if the product has reduced levels in more compounds at the same time, especially if the flavor intensity is kept at the same level, or can even be improved. This can be explained by the considerable difference between the negative consumer expectations (flavorless) and the perceived (experienced) palatability.

Similar to liking, respondents were also asked to assess the expected and perceived saltiness (salt intensity) of each type of cheese (Table 2). The internal consistency reliability of the scales was around 0.6 in all the cases, or even exceeds that (Cronbach’s alpha before tasting: 0.737, after tasting: 0.588 and before and after tasting together: 0.662).

According to expectations, the salt intensity of the product with 20% reduced salt was given the lowest mean, followed by the hybrid product, and the cheese with 20% reduced fat and 20% reduced salt. Similarly to the findings of our previous assessments, the mean of the conventional cheese was outstanding, therefore this type was considered by consumers the most salty. The mean of 4.5 probably represents a positive preference, meaning that this product meets respondents’ expectations best.

Compared to the expected salt intensity, no remarkable difference in means was observed in the assessment of perceived salt intensity (Table 2, Figure 2b). The cheese with 20% reduced fat and 20% reduced salt was evaluated the least salty, and the conventional cheese was found the most salty, with the reduced salt variant following close behind this time. This can also be attributed to the difference between the negative expectations and the experienced palatability.

The expected and perceived fat flavor intensity were also measured in the study (Table 3). The value of Cronbach’s alpha before tasting was lower: 0.466 than after tasting: 0.678, and before and after tasting together: 0.582. The higher value after tasting here also suggests that due to sensory perception, respondents were able to assess each property in a much more reliable manner.

Results indicate that the conventional cheese and the cheese with 20% reduced salt were found to be fattier in flavor, while the other two varieties were assessed as less fat flavor intensive. The results showed a substantially similar trend after tasting, as well (Table 3, Figure 2c).

Finally, we examined the expected and the actual willingness to purchase each product variant (Table 4). The values of Cronbach’s alpha were around 0.6 or above, the highest of all (Cronbach’s alpha before tasting: 0.578, after tasting: 0.723, before and after tasting together: 0.663). These findings also indicate that after tasting respondents were able to judge more reliably whether they would like to purchase the product or not.

The ranking based on willingness to purchase basically corresponds to the ranking according to expected liking. Respondents would be most willing to purchase the conventional Trappista cheese, and next in the line was the cheese with 20% reduced fat. The purchasing affinity for the cheese with 20% reduced fat and 20% reduced salt was found to be slightly higher than for the variant reduced in salt. According to the findings, the bottleneck was reduced salt content, i.e., it is this feature that proved to have the largest (negative) impact on willingness to purchase.

The order of preference changed slightly after tasting. The conventional cheese was followed by the one with 20% reduced fat and 20% reduced salt. Respondents would be more willing to purchase the cheese with 20% less fat than the variant reduced in salt (Table 4, Figure 2d).

Next, in order to make the results more refined, Scheirer–Ray–Hare (SRH) tests were performed to explore whether there are differences in the assessment made by the participants related to the four measured variables (liking, salt intensity, fat flavor intensity, willingness to purchase) between the two factors (before and after tasting, different labels) and the interaction of the two factors (time-label interaction; Table 5).

The results showed no significant differences between the assessments before and after tasting concerning any variable; it was only willingness to purchase where slightly higher (yet not significant) values were found (SS/MS = 2.069). When assessed solely on the basis of the labels, the SHR test resulted in significant differences (*p* < 0.001) according to liking, salt intensity, fat flavor intensity and willingness to purchase alike, thus confirming previous findings. The interaction of the two factors (time-label) was not found to be significant either, that is the difference in assessment before and after tasting did not tend to be related to the type of label. Effect size estimation is an essential tool to assess the strength of a statistically significant influence. It can also be seen from Table 5 that all significant effects had an average magnitude. The time factor (before and after tasting influence) had no significant effect on any factor and the effect sizes were also rather small.

Differences were detected only in some cases, which were demonstrated by using a Wilcoxon test (Table 6.).

The results of the Wilcoxon test indicate that only the reduced fat label resulted in significantly lower willingness to purchase (Z = −2.352; *p* = 0.019), and the salt intensity of the product with the conventional label was rated lower (Z = −2.231; *p* = 0.026). These results confirmed our expectations that the assessment of the sensory properties (salt and fat content) and the related expected liking of foods were influenced by preformed expectations based on health claims. When provided with the label indicating reduced salt and fat content, the palatability of the cheese, identical in composition to the others, was given a lower rating, therefore subjective prejudice had an effect on evaluation. The health-halo effect could be observed, based on which the health promoting variants were automatically associated with worse palatability and as a result, no significant changes could be detected, either in liking or in willingness to purchase even after the tasting.

## 4. Discussion and Conclusions

Based on the findings of our research we can agree with Verbeke (2006) on the fact that consumers tend to be increasingly convinced that there is no trade-off between good taste and healthiness, i.e., they are not substitutable for each other [46]. The decrease in fat and salt content is perceived by consumers to be at the expense of flavor, which they are not willing to compromise on for health (inter alia [44,45,51,52,53,54]). This finding was also confirmed by the research we conducted; reduced salt and reduced fat labels created the impression of a “flavorless” product, and respondents in the study associated them with a lack of flavor.

Studies have shown that not only do health-related claims influence health perception, but they also tend to have an impact on the liking of foods [60,65]. Our results suggest that the expectations regarding liking cheese were clearly worsened by the health claims, particularly the ones referring to salt reduction and also to salt and fat reduction. Partly similar conclusions were drawn by Schouteten et al. (2015), when conducting a cheese-experiment on a Belgian sample, where identical cheese products with different labels were tested, too [66]. Similarly to our findings, the cheese labeled “light” was given a lower mean rating by consumers, both in terms of expected and perceived liking, than the traditional control variant, despite the identical composition. The lowest liking in Belgium was for cheese labeled “light and reduced in salt”, which our research study results are partly consistent with. However, no significant differences were found between the actual, perceived liking of the conventional product and that of the cheese product reduced in salt, which corresponds to the findings by Czarnacka-Szymani and Jezewska-Zychowicz (2015) [71], while in our case salt reduction resulted in the greatest resistance. Nevertheless, both the findings by Schouteten et al. (2015) and our own findings are the same in showing that “reduction” by itself elicited the same associations from consumers in both countries. The partly different results can primarily be attributed to the differing research methods and cultural background, however the basic directions are the same [66].

Some research studies that used other food products to examine the impact of health labels on liking and flavor perception have come to similar conclusions. The expectations relating to liking were decreased both by the label indicating salt reduction in the study of Liem et al. (2012a,b) experimenting with soup, and the labels indicating fat reduction in the studies of Norton et al. (2013) and Kähkönen (2000) examining chocolate products [60,61,64,65].

Several research studies in the international literature prove that preformed expectations based on health claims on product labels influence the assessment of the sensory properties of different food products, be they a reduction in fat, reduction in salt or some other health claims (inter alia [58,61,62,63,64,65,66]). These results have been confirmed by the research we conducted, since the rating of certain properties (such as salt intensity and fat flavor intensity) after tasting showed only slight differences as compared to the ratings given before tasting, although cheese products of identical composition were tasted. Similarly to the findings of the present study, the rating of salt intensity did not change significantly, or even became lower after the actual tasting in the study conducted by Liem et al. (2012b) [61]. In Kähkönen’s experiment (2000), claims relating to fat reduction reduced the scores of sensory assessment in chocolate; however, in the research of Norton et al. (2013) it was not confirmed, since in their study, labeling did not influence the assessment of sensory properties [64,65].

The present research has also confirmed the international experience that emotional associations linked to products with different labels are also influenced by negative expectations (inter alia [66,67]). While conventional Trappista cheese was associated with traditional, nostalgic and positive images, the attitudes towards the reduced variants were mostly skeptical, and even negative in a number of cases (e.g., the product with reduced salt). However, our association map (Figure 1) confirmed that in the consumers’ minds the reduced variants were linked to the feeling of healthiness, based on which the adjective “good” was associated with this product, too.

To sum up, based on conclusions drawn from the results, there is a double approach to the evaluation of the health promoting products before tasting. On the one hand, they—especially the variants reduced in fat—are associated with a “healthy” image. On the other hand, however, consumers felt that something was taken away from them with the reductions, namely flavor, a feature they were strongly attached to. This is especially critical in the case of products reduced in salt, and of products reduced in salt and fat. After tasting, the ranking changed in several cases; however, it can be clearly concluded that the reduction in salt content can be regarded as the critical point in product development; consumers insist on the usual salt content more strongly than fat content. Naturally, all these affect willingness to purchase, too; the majority of consumers would rather buy conventional Trappista cheese, while from the health promoting variants they would be willing to purchase primarily the one with reduced fat.

Another important conclusion from the research is that—to a lesser or greater extent—differences were perceived by consumers in terms of liking and also salt and fat content, despite the fact that the samples were identical in composition, thus identical in palatability. Findings have confirmed that because of the automatic processes of human thinking, consumers are limited in their ability to make rational decisions owing to behavioral distortions, so they follow an irrational pattern of behavior. This is the so-called health-halo effect, meaning that consumers automatically associate health promoting products with less palatability and enjoyment, particularly in the case of “reduced” and “free from” versions. Participants in the study relied, without hesitation on their subjective feelings and prejudices to evaluate the palatability and the fat and salt flavor intensity of each product variant, i.e., their preformed expectations distorted the actual perception of flavor. This proved to be manifested in reduced willingness to purchase the health promoting products. To conclude, the use of evaluative labels by the producers resulted in exactly the opposite of the intended effect.

It should be pointed out as a limitation of our survey that the experiment was of an exploratory nature with a small sample size, and it was intended to develop methodology. By presenting quantitative data and statistical analyses we aimed to demonstrate differences and deviations. Based on all these, the results cannot be generalized; however, since the methodology of the experiment has proved to be suitable to demonstrate differences, future surveys on representative samples are desirable.

## Figures and Tables

**Table 1:** The expected pretasting and perceived post-tasting liking of the four types of Trappista cheese (*N* = 32).

Product	Expected Liking Before Tasting					Perceived Liking After Tasting				
	Mean	Median	Mode	Standard Deviation	Relative Standard Deviation (%)	Mean	Median	Mode	Standard Deviation	Relative Standard Deviation (%)
Conventional cheese	5.66	6.00	5.00	0.94	16.57	5.53	5.50	5.00	1.11	20.01
Cheese with 20% lower salt content	4.31	4.00	4.00	1.09	25.29	4.28	4.50	5.00	1.37	32.08
Cheese with 20% lower fat content	4.78	5.00	4.00	1.18	24.77	4.69	5.00	5.00	1.26	26.79
Cheese with 20% lower fat and 20% lower salt content	4.29	4.00	4.00	1.55	36.21	4.84	5.00	5.00	1.44	29.71

**Table 2:** The expected, pretasting and the perceived, post-tasting salt intensity of the four types of Trappista cheese (*N* = 32).

Product	Expected Salt Intensity before Tasting					Perceived Salt Intensity after Tasting				
	Mean	Median	Mode	Standard Deviation	Relative Standard Deviation (%)	Mean	Median	Mode	Standard Deviation	Relative Standard Deviation (%)
Conventional cheese	4.50	5.00	5.00	0.88	19.55	3.97	4.00	4.00	0.97	24.36
Cheese with 20% lower salt content	3.48	3.00	4.00	1.34	38.42	3.81	4.00	3.00	1.40	36.76
Cheese with 20% lower fat content	4.00	4.00	3.00	1.14	28.40	3.66	3.50	3.00	0.94	25.63
Cheese with 20% lower fat and 20% lower salt content	3.50	3.00	3.00	1.37	39.08	3.56	4.00	3.00	1.13	31.84

**Table 3:** The expected pretasting, and the perceived post-tasting fat flavor intensity of the four types of Trappista cheese (*N* = 32).

Product	Expected Fat Flavor Intensity before Tasting					Perceived Fat Flavor Intensity after Tasting				
	Mean	Median	Mode	Standard Deviation	Relative Standard Deviation (%)	Mean	Median	Mode	Standard Deviation	Relative Standard Deviation (%)
Conventional cheese	4.59	5.00	5.00	1.19	25.85	4.66	5.00	5.00	1.04	22.23
Cheese with 20% lower salt content	4.03	4.00	4.00	0.97	23.98	4.03	4.00	4.00	0.97	23.98
Cheese with 20% lower fat content	3.41	3.00	3.00	1.24	36.42	3.66	4.00	4.00	1.10	29.97
Cheese with 20% lower fat and 20% lower salt content	3.44	3.00	3.00	1.29	37.63	3.44	4.00	4.00	1.19	34.61

**Table 4:** The expected pretasting and the perceived post-tasting willingness to purchase the four types of Trappista cheese (*N* = 32).

Product	Expected Willingness to Purchase before Tasting					Perceived Willingness to Purchase after Tasting				
	Mean	Median	Mode	Standard Deviation	Relative Standard Deviation (%)	Mean	Median	Mode	Standard Deviation	Relative Standard Deviation (%)
Conventional cheese	5.28	5.00	5.00	1.11	21.09	5.06	5.00	5.00	1.08	21.25
Cheese with 20% lower salt content	4.34	4.00	4.00	1.12	25.89	3.97	4.00	3.00	1.40	35.34
Cheese with 20% lower fat content	4.78	5.00	5.00	1.36	28.48	4.22	4.00	5.00	1.43	33.92
Cheese with 20% lower fat and 20% lower salt content	4.41	4.00	4.00	1.62	36.85	4.53	4.50	3.00	1.63	35.89

**Table 5:** Scheirer–Ray–Hare (SRH) test based on two factors (before and after tasting, different labels) and the interaction of these two factors (*N* = 32).

Factor		SS Liking/MS Liking	SS Salt Intensity/MS Salt Intensity	SS Fat Flavor Intensity/MS Fat Flavor Intensity	SS Willingness to Purchase/MS Willingness to Purchase
Assessment before and after tasting	SRH test	0.526	0.5102	0.427	2.069
	Freeman’s theta	0.051	0.050	0.046	0.101
Labels with different information	SRH test	38.357 ***	17.034 ***	40.298 ***	19.056 ***
	Freeman’s theta	0.304	0.202	0.305	0.215
Time-label interaction	SRH test	3.002	5.057	0.402	2.075
	Freeman’s theta	0.285	0.205	0.289	0.214

Notes: * *p* < 0.05; ** *p* < 0.01; *** *p* < 0.001; SS = sum of squares; MS = mean square; Freeman’s theta statistic was given for effect size calculation. Average and acceptable effect sizes lie within the range of 0.2 and 0.4.

**Table 6:** Results of the Wilcoxon test (Z-statistics) to compare the assessments before and after tasting (*N* = 32).

Z-Statistics	Liking	Salt Intensity	Fat Flavor Intensity	Willingness to Purchase
Conventional cheese found in shops	−0.463	−2.231 *	−0.146	−0.687
Cheese with 20% lower salt content	−0.413	−1.124	−0.125	−1.349
Cheese with 20% lower fat content	−0.116	−1.383	−1.138	−2.352 *
Cheese with 20% lower fat and 20% lower salt content	−1.399	−0.026	−0.067	−0.05

Note: * *p* < 0.05.

## Appendix A. Test Sheet I

1 Before tasting, tell us what is your first association generated by each version of the product?

**2 Before tasting, to what extent do you think you will like them?**

**Table A1:** Conventional cheese.

Extremely Dislike						Extremely Like
1	2	3	4	5	6	7

**Table A2:** Reduced-sodium cheese.

Extremely Dislike						Extremely Like
1	2	3	4	5	6	7

**Table A3:** Reduced-fat cheese.

Extremely Dislike						Extremely Like
1	2	3	4	5	6	7

**Table A4:** Reduced fat and reduced-sodium cheese.

Extremely Dislike						Extremely Like
1	2	3	4	5	6	7

**3 Before tasting, to what extent do you think each version of the product will be salty?**

**Table A5:** Conventional cheese.

Not Salty at All						Extremely Salty
1	2	3	4	5	6	7

**Table A6:** Reduced-sodium cheese.

Not Salty at All						Extremely Salty
1	2	3	4	5	6	7

**Table A7:** Reduced-fat cheese.

Not Salty at All						Extremely Salty
1	2	3	4	5	6	7

**Table A8:** Reduced fat and reduced-sodium cheese.

Not Salty at All						Extremely Salty
1	2	3	4	5	6	7

**4 Before tasting, to what extent do you think each version of the product will be fatty?**

**Table A9:** Conventional cheese.

Not Fatty at All						Extremely Fatty
1	2	3	4	5	6	7

**Table A10:** Reduced-sodium cheese.

Not Fatty at All						Extremely Fatty
1	2	3	4	5	6	7

**Table A11:** Reduced-fat cheese.

Not Fatty at All						Extremely Fatty
1	2	3	4	5	6	7

**Table A12:** Reduced fat and reduced-sodium cheese.

Not Fatty at All						Extremely Fatty
1	2	3	4	5	6	7

**5 Before tasting, to what extent do you think you will be willing to purchase each version of the product?**

**Table A13:** Conventional cheese.

Not Willing to Purchase at All						Extremely Willing to Purchase
1	2	3	4	5	6	7

**Table A14:** Reduced-sodium cheese.

Not Willing to Purchase at All						Extremely Willing to Purchase
1	2	3	4	5	6	7

**Table A15:** Reduced-fat cheese.

Not Willing to Purchase at All						Extremely Willing to Purchase
1	2	3	4	5	6	7

**Table A16:** Reduced fat and reduced-sodium cheese.

Not Willing to Purchase at All						Extremely Willing to Purchase
1	2	3	4	5	6	7

## Appendix B. Test Sheet II

**1 After tasting, how much do you like each version of the product?**

**Table A17:** Conventional cheese.

Extremely Dislike						Extremely Like
1	2	3	4	5	6	7

**Table A18:** Reduced-sodium cheese.

Extremely Dislike						Extremely Like
1	2	3	4	5	6	7

**Table A19:** Reduced-fat cheese.

Extremely Dislike						Extremely Like
1	2	3	4	5	6	7

**Table A20:** Reduced fat and reduced-sodium cheese.

Extremely Dislike						Extremely Like
1	2	3	4	5	6	7

**2 After tasting, to what extent do you find each version of the product salty?**

**Table A21:** Conventional cheese.

Not Salty at All						Extremely Salty
1	2	3	4	5	6	7

**Table A22:** Reduced-sodium cheese.

Not Salty at All						Extremely Salty
1	2	3	4	5	6	7

**Table A23:** Reduced-fat cheese.

Not Salty at All						Extremely Salty
1	2	3	4	5	6	7

**Table A24:** Reduced fat and reduced-sodium cheese.

Not Salty at All						Extremely Salty
1	2	3	4	5	6	7

**3 After tasting, to what extent do you find each version of the product fatty?**

**Table A25:** Conventional cheese.

Not Fatty at All						Extremely Fatty
1	2	3	4	5	6	7

**Table A26:** Reduced-sodium cheese.

Not Fatty at All						Extremely Fatty
1	2	3	4	5	6	7

**Table A27:** Reduced-fat cheese.

Not Fatty at All						Extremely Fatty
1	2	3	4	5	6	7

**Table A28:** Reduced fat and reduced-sodium cheese.

Not Fatty at All						Extremely Fatty
1	2	3	4	5	6	7

**4 After tasting, to what extent would you be willing to purchase each version of the product?**

**Table A29:** Conventional cheese.

Not Willing to Purchase at All						Extremely Willing to Purchase
1	2	3	4	5	6	7

**Table A30:** Reduced-sodium cheese.

Not Willing to Purchase at All						Extremely Willing to Purchase
1	2	3	4	5	6	7

**Table A31:** Reduced-fat cheese.

Not Willing to Purchase at All						Extremely Willing to Purchase
1	2	3	4	5	6	7

**Table A32:** Reduced fat and reduced-sodium cheese.

Not Willing to Purchase at All						Extremely Willing to Purchase
1	2	3	4	5	6	7
